# The complete mitochondrial genome of *Montiporavietnamensis* (Scleractinia, Acroporidae)

**DOI:** 10.3897/BDJ.10.e91531

**Published:** 2022-09-13

**Authors:** Wei Wang, Bingbing Cao, Ziqing Xu, Zhiyu Jia, Shuangen Yu, Peng Tian, Wentao Niu, Jiaguang Xiao

**Affiliations:** 1 Third Institute of Oceanography, Ministry of Natural Resources, Xiamen, China Third Institute of Oceanography, Ministry of Natural Resources Xiamen China; 2 Key Laboratory of Mariculture of Ministry of Education, College of Fisheries, Ocean University of China, Qingdao, China Key Laboratory of Mariculture of Ministry of Education, College of Fisheries, Ocean University of China Qingdao China

**Keywords:** mitochondrial genome, primers, Acroporidae, *Montiporavietnamensis*

## Abstract

*Montiporavietnamensis* Veron, 2000 (Cnidaria, Anthozoa, Scleractinia, Acroporidae) is an uncommon, but distinctive species of stony coral. The complete mitochondrial genome of *M.vietnamensis* was sequenced in this study for the first time, based on 32 pairs of primers newly designed according to seven species in the family Acroporidae. The mitogenome of *M.vietnamensis* has a circular form and is 17,885 bp long, including 13 protein-coding genes (PCGs), 2 tRNA (tRNA^Met^, tRNA^Trp^), 2 rRNA genes and a putative control-region. The base composition of the complete mitogenome was 24.8% A, 14.2% C, 24.2% G and 36.8% T, with a higher AT content (61.6%) than GC content (38.4%). Based on 13 protein-coding genes, a Maximum Likelihood phylogenetic analysis showed that *M.vietnamensis* is clustered in the genus *Montipora* which belongs to the family Acroporidae. More stony coral species should be sequenced for basic molecular information and to help confirm the taxonomic status and evolutionary relationships of Scleractinia in the future.

## Introduction

Reef-building coral species of the order Scleractinia play an important role in shallow tropical seas by providing an environmental foundation for the ecosystem (Fukami et al. 2000, Sheppard et al. 2017). While traditional morphology-based systematics cannot clearly reflect all the evolutionary relationships of Scleractinia, molecular data have become increasingly important in recent years to help overcome the limitations of morphological analyses amongst scleractinians (Arrigoni et al. 2017, Terraneo et al. 2017).

Cnidarian mitogenome data contain important phylogenetic information for understanding its evolutionary history (Kayal et al. 2013). The utility of integrating morphological and genetic datasets also facilitates the taxonomic revisions of scleractinian taxa (Juszkiewicz et al. 2022). There are more than 1600 Scleractinia species, whereas only approximately 100 complete mitogenomes of Scleractinia species are currently available in NCBI (https://www.ncbi.nlm.nih.gov/) (Hoeksema and Cairns 2022).

*Montiporavietnamensis* Veron, 2000 (Cnidaria, Anthozoa, Scleractinia, Acroporidae) is a species of stony coral, which is uncommon, but distinctive and usually inhabits shallow reef environments and rocky foreshores. Its colonies have an encrusting or laminar base, with closely compacted short upright branches; their coenosteum ridges are mostly vertical, but may be irregular; their corallites are large and prominent and their colours are dark brown, usually with white coenosteum ridges and branch tips (Veron 2000).

In this research, the complete mitochondrial genome of *M.vietnamensis* was sequenced for the first time, based on 32 pairs of primers designed according to seven species in the family Acroporidae. The phylogenetic position of *M.vietnamensis* within the family Acroporidae, based on protein coding genes of the mitogenome, will help determine its taxonomic status and facilitate further study on stony coral evolutionary and phylogenetic relationships (Tian et al. 2022). Ultimately, this information can aid in species monitoring and conservation efforts (Colin et al. 2021).

## Material and methods

**Figure 1.**
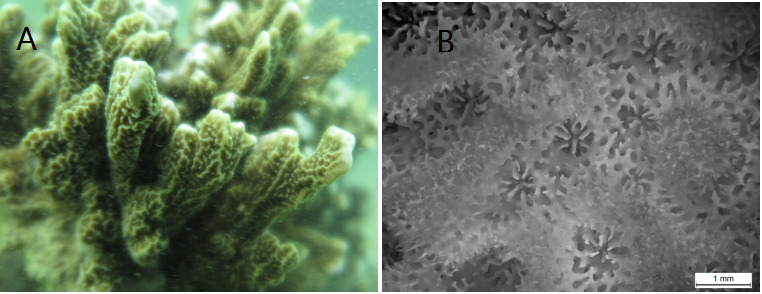
Photos of *M.vietnamensis* examined in this study. **A** In-situ photograph of *M.vietnamensis*; **B** Microskeletal photograph of *M.vietnamensis*.

Two samples of *M.vietnamensis* (Fig. 1) were collected from Houhai, Sanya, Hainan Province, China (109°44' 55.91"E, 18°16' 28.58" N); one of them was immediately placed in a single vial in ethanol (+99%) and labelled with a unique identifier E38. This sample was then stored at -20°C until extraction. The other one was bleached by soaking in 5% sodium hypochlorite and then the specimen was kept in our Coral Sample Repository with a special code, 20181124-E38 (contact the first author to view or loan this specimen). Species identification was conducted according to the photographs and description of Veron (2000)(http://www.coralsoftheworld.org/species_factsheets/species_factsheet_summary/montipora-vietnamensis/). Complete genomic DNA (gDNA) was extracted using the DNeasy Blood and Tissue Kit (Qiagen, Shanghai, China), following the protocol at https://www.qiagen.com/cn/resources/resourcedetail?id=68f29296-5a9f-40fa-8b3d-1c148d0b3030&lang=en. Electrophoresis with 1% agarose gel was used to estimate the integrity of the gDNA and the spectrophotometer NanoDrop 2000 (Thermo Scientific, USA) was used to measure the gDNA concentration.

The mitogenome sequence fragments were obtained through a PCR approach using 32 pairs of primers (Table 1) designed through primer-blast (https://www.ncbi.nlm.nih.gov/tools/primer-blast/), based on seven Acroporidae species that had been sequenced and data available in https://www.ncbi.nlm.nih.gov/genbank/ (NC_029251, KF448533, C_024092, NC_040137, MG851913, KJ634269, NC_006902). The PCR used 25 μl mixtures containing 2.5 μl of 10x ExTaq Buffer (20 mM), 2 μl dNTP, 1 μl of each primer(10 μM), 0.13 μl ExTaq DNA polymerase (Takara Product Code No. RR001Q, Beijing, China) and approximately 0.5 μg of gDNA. Cycling conditions consisted of 5 min at 95°C; then 30 cycles of 30 s at 95°C, 45 s at 50°C and 1 min at 72°C; followed by a final extension at 72°C for 10 min. The PCR products were directly sequenced using an ABI 3730XL automated DNA sequencer (Applied Biosystems, Sangon Biotech, Shanghai, China). We assembled all the sequencing fragments as a circularised contig using ContigExpress v.3.0.0. The circularised contig was then submitted to MITOS (Bernt et al. 2013) WebServer (http://mitos.bioinf.uni-leipzig.de/index.py) for preliminary mitochondrial genome annotation. We then identified and annotated the 13 PCGs and RNA genes by alignments of homologous mitogenomes of other scleractinians that had been uncovered through BLAST searches in NCBI (https://blast.ncbi.nlm.nih.gov/Blast.cgi). The genomic structure was mapped using the online CGView Server (https://proksee.ca/) (Stothard and Wishart 2004).

The phylogenetic position of *M.vietnamensis* within the family Acroporidae was inferred using 13 tandem mitogenome PCG sequences, with 19 of the other 21 species of Scleractinia analysed in this study obtained from GenBank (https://www.ncbi.nlm.nih.gov/genbank/, Table 2). Two other species, *Acroporadigitifera* (GenBank accession number: OP311587) and *Acroporahyacinthus* (GenBank accession number: OP311657), were sequenced using the same primers as *M.vietnamensis*. We used MEGA 7 (Kumar et al. 2016) to select the best-fitting model, based on the Akaike Information Criterion (AIC) and then constructed a Maximum Likelihood (ML) tree with 500 bootstrap replicates.

## Results and Discussion

**Figure 2.**
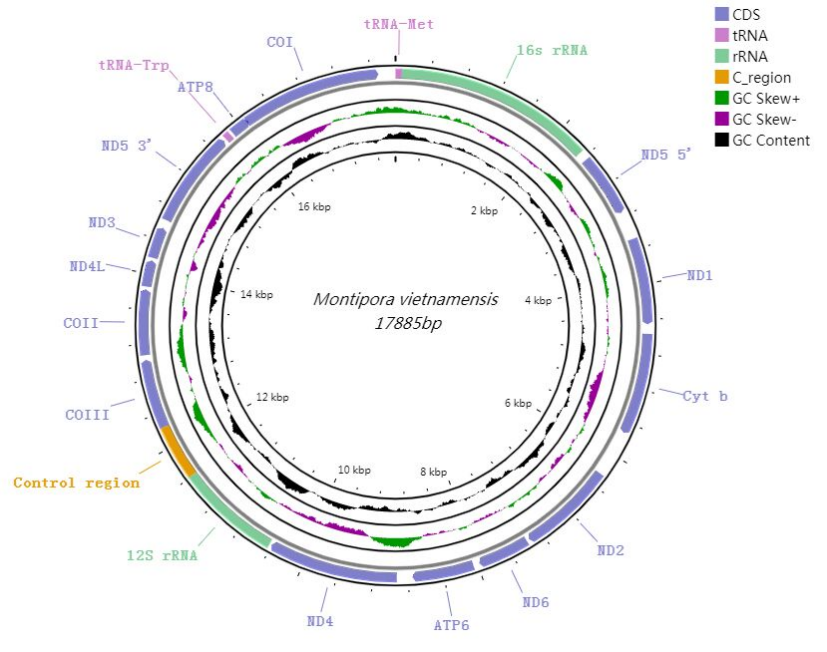
The mitochondrial genome of *M.vietnamensis*. Gene order and positions are shown. COI, COII and COIII refer to the cytochrome oxidase subunits, Cyt b refers to cytochrome b and ND1-ND6 refers to NADH dehydrogenase components. All genes are encoded on the H-strand.

The mitochondrial genome size of *M.vietnamensis* (GenBank accession number: ON872180, https://www.ncbi.nlm.nih.gov/nucleotide) was 17,885 bp, including 13 PCGs, 2 tRNA (tRNAMet, tRNATrp), 2 rRNA genes and a putative control-region (Fig. 2, Table 3). The mitogenome of *M.vietnamensis* offered no distinct structure and its gene order was the same as those of published mitogenomes of Acroporidae species, with all genes encoded on the H-strand. The base composition of the complete mitogenome was 24.8% A, 14.2% C, 24.2% G and 36.8% T, with a higher AT content (61.6%) than GC content (38.4%). The total length of all 13 PCGs was 11,817 bp, with a base composition of 22.1%, 14.5%, 23.7% and 39.7% for A, C, G and T, respectively. ND5 gene had an intron insertion of 11,489 bp. The shortest gene was ATP8 (218 bp) and the longest gene was ND5 (1,836 bp). The putative control-region was 627 bp (Tables 3, 4).

The encoding genes 12S rRNA and 16S rRNA in *M.vietnamensis* were 1,175 bp and 2,260 bp in size, respectively. Both the two rRNAs’ base composition was 32.5% A, 14.5% C, 25.5% G and 27.5% T. The two tRNA encoding genes tRNA^Met^ and tRNA^Trp^ were 71 bp and 70 bp in size, respectively.

**Figure 3.**
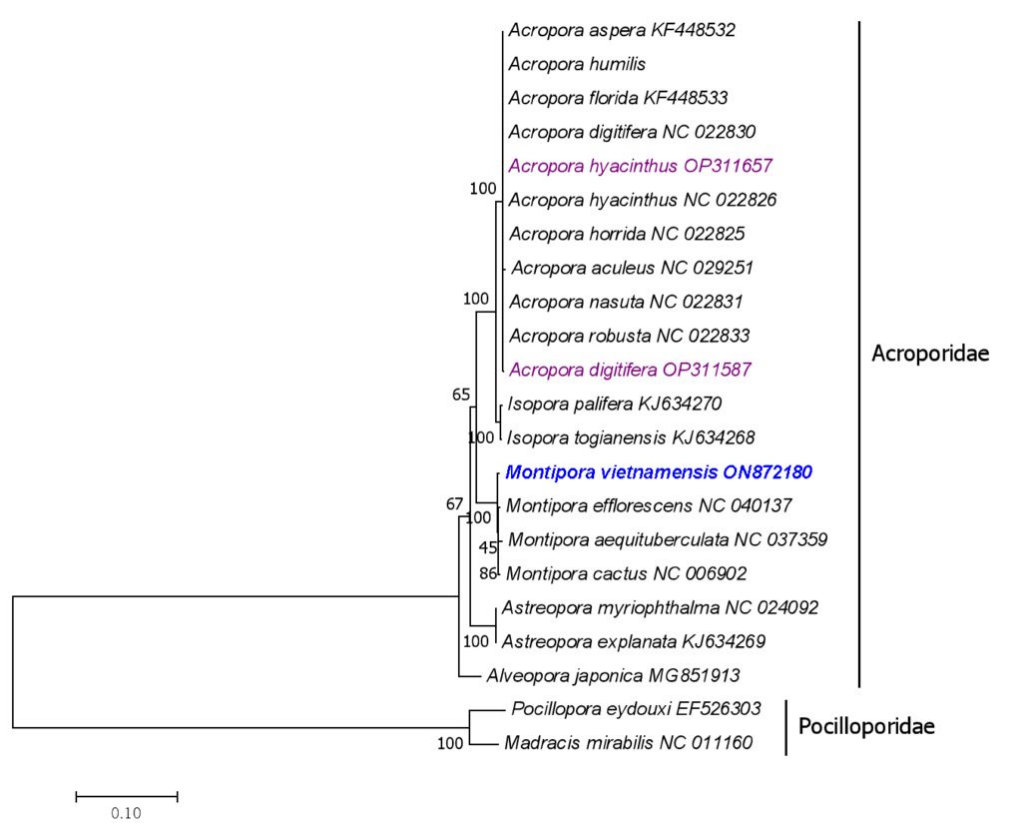
Inferred phylogenetic relationships, based on a Maximum-Likelihood analysis of concatenated nucleotide sequences of 13 mitochondrial PCGs. Numbers on branches are bootstrap percentages.

The ML bootstrap consensus tree shows that *M.vietnamensis* is clustered in the genus *Montipora* which belongs to the family Acroporidae with high bootstrap support (Fig. 3). The mitochondrial genome data have provided important molecular information for understanding evolutionary relationships amongst stony corals (Kitahara et al. 2016, Arrigoni et al. 2020). In this research, the 32 pairs of primers we designed according to seven Acroporidae species comprised a useful tool to obtain the mitogenome of *M.vietnamensis*. With the same primer sets, we further obtained four mitogenomes of other Acroporidae species, *Acroporadigitifera* (GenBank accession number: OP311587), *Acroporahyacinthus* (GenBank accession number: OP311657), *Acroporaintermedia* (GenBank accession number: OP311588) and *Acroporamicrophthalma* (GenBank accession number: OP311656). These showed 99.82%, 99.99%, 99.79% and 99.98% sequence identity with conspecifics already sequenced and available in GenBank that were obtained by next-generation sequencing (NGS). The NGS method was convenient, fast and relatively accurate. However, it cost less and was more time-efficient when we sequenced these five samples using the current Sanger sequencing approach. More stony coral species should be sequenced for basic molecular information and to help confirm the taxonomic status and evolutionary relationships of Scleractinia in the future.

## Figures and Tables

**Table 1.** Total of 32 pairs of primers designed, based on seven Acroporidae species.

No.	Name	primer sequences
1	Acro16SF1	5'-ATTCCGTAAGTAGCAGGGAG-3'
2	Acro16SR1	5'-TTGTCTAAATCCCATACTCC-3'
3	Acro16SF2	5'-TTCGAAGTAGACAGACAGAC-3'
4	Acro16SR2	5'-GCAGGTCTCACCCTTCATAC-3'
5	Acro16SF3	5'-TAAGGAACTCGGCCAGTTAT-3'
6	Acro16SR3	5'-GACGTTATTACGCTGTTATC-3'
7	Acro16SF4	5'-GAGCAGACACTTATCTTTGG-3'
8	Acro16SR4	5'-CTTATAATCAAACAGCTTGAAG-3'
9	AcroND5F5	5'-GTTGGAGGAAGAAAATTAGG-3'
10	AcroND5R5	5'-AGCCCCAACTGTGCAGACTT-3'
11	AcroND5F6	5'-GGGTCTTTAGAGTTTTCTTC-3'
12	AcroND5R6	5'-CTTCATAACTAATCATTTGAGC-3'
13	AcroND1F7	5'-GGCTGTTTCTTCGATAAGTG-3'
14	AcroND1R7	5'-ACGCCTTTCATAACAAAGAC-3'
15	AcroND1F8	5'-GCCTCTTTTCCTCGTATTCG-3'
16	AcroND1R8	5'-CTCAAGGTAGCACATAGCCC-3'
17	AcroCytbF9	5'-CCGGTTTGGCGAGTTGGCAT-3'
18	AcroCytbR9	5'-CGTCCAAATGGACGAAAGGG-3'
19	AcroCytbF10	5'-GCACATTCAGCCTGAGTGAT-3'
20	AcroCytbR10	5'-CTCCCGTAAACCCACACAAT-3'
21	AcroND2F11	5'-CTTCAAGTAATTGACTTTCTG-3'
22	AcroND2R11	5'-ACCTCTATTCCCCAAAGCAC-3'
23	AcroND2F12	5'-TTGGGGCTCTTTTTTCGATG-3'
24	AcroND2R12	5'-CCAATAACATACAAACCAGC-3'
25	AcroND2F13	5'-CTCTTTTGATAAGCTCAAAG-3'
26	AcroND2R13	5'-CCCAATAGGAATGTAATTTGTC-3'
27	AcroND6F14	5'-CGCTCAATCCTATCCATTCG-3'
28	AcroND6R14	5'-CCCAATTTCTTGAGTTAACAC-3'
29	AcroND6F15	5'-GCGAATTTTGTATATAGCTTG-3'
30	AcroND6R15	5'-CCAAACCCGGCTAAAATAGC-3'
31	AcroATP6F16	5'-GTAAGTTTTATCTCCAGGGC-3'
32	AcroATP6R16	5'-TCAAGCACTAAAAACACTCC-3'
33	AcroND4F17	5'-AAGTTGAAAGTCCATTAAGC-3'
34	AcroND4R17	5'-TGTGCCACCGAAGAATAAGC-3'
35	AcroND4F18	5'-TTTTCTTGGCCGATTTTGCC-3'
36	AcroND4R18	5'-TTACCCCATTCTTTACAGGG-3'
37	AcroND4F19	5'-CTTCGGGTATGGTTTGGTCC-3'
38	AcroND4R19	5'-TGGCACTTAATTTGACGGAC-3'
39	Acro12SF20	5'-AGCCACATTTTCACTGAGAC-3'
40	Acro12SR20	5'-AAACCACTGGGTTAAATCTG-3'
41	Acro12SF21	5'-AGAGACCTTACCCAAACTTG-3'
42	Acro12SR21	5'-CTCTAATAACATCTTGTCATC-3'
43	AcroCO3F22	5'-GTTGAGCCTTCTCCTTGGCC-3'
44	AcroCO3R22	5'-AATGCCAATACCAACTCGCC-3'
45	AcroCO3F23	5'-TTTCACTATTTCGGATTCGG-3'
46	AcroCO3R23	5'-TTAAATCCGATGTCGGAACC-3'
47	AcroCO2F24	5'-GGACATCAATGGTATTGGTC-3'
48	AcroCO2R24	5'-ACCCCGAAGTGAACTAAAAG-3'
49	AcroND4LF25	5'-TTATGGGTTTAACAATCGCG-3'
50	AcroND4LR25	5'-AGCCCACCTTTAATCCACTC-3'
51	AcroND3F26	5'-TTTCTTTTCCCTTGGTGTGT-3'
52	AcroND3R26	5'-TATTGTTCAAAGGCCAATTC-3'
53	AcroND5F27	5'-TGTCATCCATGCTTTGTCTG-3'
54	AcroND5R27	5'-TTTGTCAATAGTCCGATACG-3'
55	AcroND5F28	5'-TTATTAAGTTGTTGCCGGTC-3'
56	AcroND5R28	5'-TTCTTTAGTTAGCCCCAAAC-3'
57	AcroATP8F29	5'-TTAACTCAATATCGATGAAC-3'
58	AcroATP8R29	5'-CCCAAAATCGAAGACACCCC-3'
59	AcroCO1F30	5'-CCTCTATCGAGCATCCAGGC-3'
60	AcroCO1R30	5'-CATTGCCCAAAGCATAGGAG-3'
61	AcroCO1F31	5'-CGCAACTATGATTATTGCTG-3'
62	AcroCO1R31	5'-CAACCAGCAAAACAATCTGC-3'
63	AcroCO1F32	5'-TGTTATAATGAGCTATATGG-3'
64	AcroCO1R32	5'-GCCTCTTCTTCGCTCTTTCG-3'

**Table 2.** Representative species of Scleractinia included in this study.

NO.	Species	Family	Length (bp)	GenBank accession number
1	*Montiporavietnamensis*	Acroporidae	17,885	ON872180
2	*Acroporaaculeus*	Acroporidae	18,528	NC_029251
3	*Acroporadigitifera*	Acroporidae	18,480	OP311587
4	*Acroporadigitifera*	Acroporidae	18,479	NC_022830
5	*Acroporahyacinthus*	Acroporidae	18,567	OP311657
6	*Acroporahyacinthus*	Acroporidae	18,566	NC_022826
7	*Acroporaflorida*	Acroporidae	18,365	KF448533
8	*Acroporahorrida*	Acroporidae	18,480	NC_022825
9	*Acroporanasuta*	Acroporidae	18,481	NC_022831
10	*Acroporarobusta*	Acroporidae	18,480	NC_022833
11	*Astreoporamyriophthalma*	Acroporidae	18,106	NC_024092
12	*Montiporaaequituberculata*	Acroporidae	17,886	NC_037359
13	*Montiporaefflorescens*	Acroporidae	17,886	NC_040137
14	*Acroporaaspera*	Acroporidae	18,479	KF448532
15	*Acroporahumilis*	Acroporidae	18,479	KF448528
16	*Alveoporajaponica*	Acroporidae	18,144	MG851913
17	*Astreoporaexplanata*	Acroporidae	18,106	KJ634269
18	*Isoporapalifera*	Acroporidae	18,725	KJ634270
19	*Isoporatogianensis*	Acroporidae	18,637	KJ634268
20	*Montiporacactus*	Acroporidae	17,887	NC_006902
21	*Pocilloporaeydouxi*	Pocilloporidae	17,422	EF526303
22	*Madracismirabilis*	Pocilloporidae	16,951	NC_011160

**Table 3.** Organisation of the mitochondrial genome of *M.vietnamensis*.

Sequence	Position		Size (bp)	Amino	Gaps	Codon		Strand
	From	To	Nucleotide	acid		Start	Stop	
tRNA^Met^	1	71	71		0			H
16s rRNA	72	2331	2260		102			H
ND5 5'	2434	3153	720	240	322	GTG		H
ND1	3476	4459	984	327	106	GTG	TAA	H
Cyt *b*	4566	5723	1158	385	533	ATG	TAG	H
ND2	6257	7354	1098	365	32	ATG	TAA	H
ND6	7387	7980	594	197	71	ATA	TAA	H
ATP6	8052	8750	699	232	179	ATG	TAG	H
ND4	8930	10405	1476	491	28	GTG	TAA	H
12S rRNA	10434	11608	1175		0			H
Control region	11609	12235	627		0			H
CO III	12236	13024	789	262	55	GTG	TAG	H
CO II	13080	13823	744	247	35	ATG	TAA	H
ND4L	13859	14158	300	99	31	GTG	TAA	H
ND3	14190	14546	357	118	96	GTG	TAG	H
ND5 3'	14643	15758	1116	371	29		TAG	H
tRNA^Trp^	15788	15857	70		32			H
ATP8	15890	16108	219	72	-19	ATG	TAG	H
COI	16090	17691	1602	533	194	ATG	TAA	H

**Notes**: The gaps are number of nucleotides between the given gene and the related gene behind, negative numbers indicating overlapping nucleotides; H indicated that the genes were transcribed on the heavy strand.

**Table 4.** Nucleotide composition features in *M.vietnamensis*.

Gene/Region	T%	C%	A%	G%	A+T%	size（bp）
Overall	36.8	14.2	24.8	24.2	61.6	17885
Control region	36.7	12.8	23.8	26.8	60.4	627
rRNA	27.5	14.5	32.5	25.5	60	141
tRNA	20.6	23.4	24.8	31.2	45.4	3435
PCGs	39.7	14.5	22.1	23.7	61.8	11817
1^st^	32	13.5	24.3	30.2	56.3	3939
2^nd^	45	19.9	18.4	16.7	63.4	3939
3^rd^	42.1	10.2	23.7	24	34.3	3939
